# Transcatheter Tricuspid Valve Replacement With EVOQUE and Mitral Valve Repair With PASCAL in One Procedure

**DOI:** 10.1016/j.jaccas.2024.102760

**Published:** 2025-01-15

**Authors:** Varius Dannenberg, Caglayan Demirel, Anna Bartunek, Philipp E. Bartko

**Affiliations:** a Department for Internal Medicine II, Cardiology, Medical University of Vienna, Vienna, Austria; b Department of Anaesthesiology, General Intensive Care and Pain Medicine, Division of Cardiac Thoracic Vascular Anesthesia and Intensive Care Medicine, Medical University Vienna, Vienna, Austria

**Keywords:** echocardiography, mitral valve, tricuspid valve, valve repair, valve replacement

## Abstract

Mitral and tricuspid regurgitation, linked to high morbidity and mortality, are increasingly treated with interventional edge-to-edge repair, showing excellent results in favorable anatomy. Recently, interventional valve replacement strategies have emerged. We present a patient with severe dyspnea and leg edema who was diagnosed with severe mitral and torrential tricuspid regurgitation. In one procedure, she underwent mitral edge-to-edge repair with a PASCAL Ace and transcatheter tricuspid valve replacement with an EVOQUE 48 mm (both Edwards Lifesciences). Tricuspid regurgitation was reduced to trace, and mitral regurgitation was mild and stable at 1 month of follow-up. It is still being determined whether mitral and tricuspid regurgitation should be treated simultaneously or in stages. Recent data show favorable results for simultaneous treatment, but tricuspid regurgitation may improve after mitral valve repair alone. Mitral and tricuspid regurgitation can be effectively treated in challenging anatomies with new replacement devices in a single procedure, offering patients fast and safe relief from both conditions.

## Visual Summary

**Figure:**
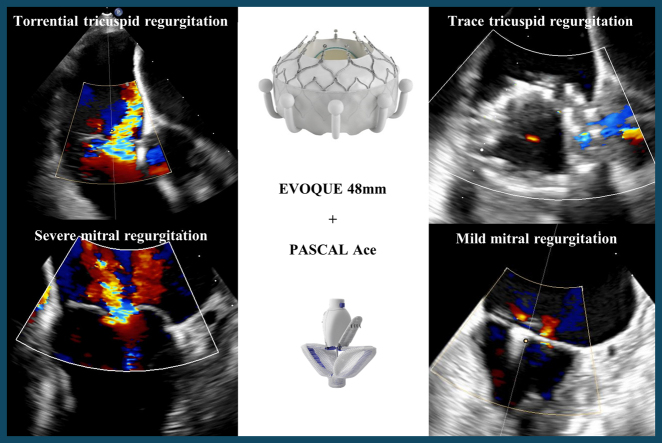

Mitral regurgitation (MR) and tricuspid regurgitation (TR) are common heart valve diseases with high morbidity and mortality.^1^^,^^2^ Interventional treatments for both are established in clinical practice and recommended by the American College of Cardiology and the American Heart Association.^3^ A recent analysis showed better outcomes for patients treated with mitral edge-to-edge repair (M-TEER) and tricuspid edge-to-edge repair (T-TEER) than M-TEER alone.^4^ Although MR is typically repaired before TR, the optimal timing for tricuspid valve repair remains unclear. A study found that TR worsened in about one-fifth of patients and remained unchanged in about half after mitral valve repair, suggesting that two-thirds might benefit from simultaneous repair of both valves.^5^

**Take-Home Messages**

• Mitral and tricuspid regurgitation can be effectively treated in difficult anatomical conditions with new replacement devices and established edge-to-edge repair therapy.
• In carefully selected instances (anatomical and clinical), simultaneous treatment of MR and TR can be done safely.

The most common method for repairing MR and TR is edge-to-edge repair using the PASCAL system (Edwards Lifesciences) or the MitraClip/TriClip (Abbott). These procedures show excellent results in favorable anatomy but may be inadequate in complex cases.^6^^,^^7^ Several unfavorable criteria, such as a large gap or restricted or calcified leaflets, can negatively affect the long-term effectiveness of the procedure and may lead to single-leaflet device attachments. For complex valve conditions, the EVOQUE (Edwards Lifesciences) transcatheter tricuspid valve replacement system (TTVR) is a suitable alternative to T-TEER, effectively treating tricuspid regurgitation via a transcatheter femoral approach.^8^

We present a case report of a patient with MR and TR treated with a PASCAL precision system on the mitral side and an EVOQUE on the tricuspid side within 1 procedure (Video 1).

## History of Presentation

We present an 80-year-old female patient with severe dyspnea and marked leg edema. She was treated with diuretic agents in an outpatient clinic for several months but was now experiencing progressive symptoms.

## Past Medical History

The patient has known heart failure with preserved ejection fraction, atrial fibrillation, arterial hypertension, chronic obstructive pulmonary disease, and hypothyroidism.

## Investigations

An electrocardiogram showed atrial fibrillation with a QRS duration of 96 ms. Transthoracic echocardiography revealed torrential functional atrial TR and severe primary MR caused by a confined prolapse of the P2 segment. Right and left ventricular function were preserved. Invasive hemodynamics revealed mild postcapillary pulmonary hypertension and a coronary angiogram ruled out significant coronary artery stenosis.

## Management

**Figure 1:**
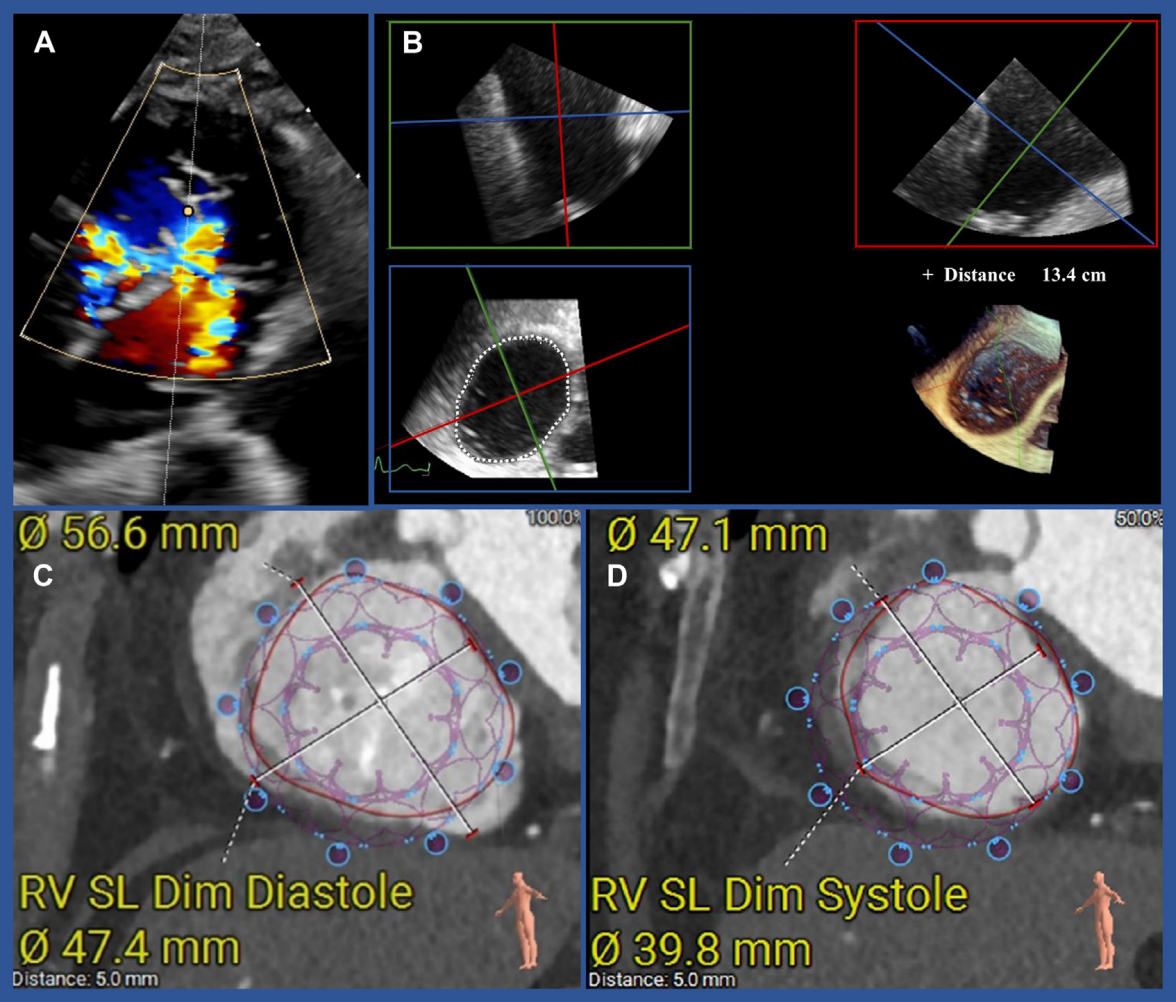
Screening for Transcatheter Tricuspid Valve Replacement (EVOQUE) (A) Torrential tricuspid regurgitation according to echocardiography. (B) Sizing the tricuspid valve using echocardiography and multiplane reconstruction. (C and D) Sizing the tricuspid valve using computed tomography. RV SL Dim = right ventricular septal lateral diameter.

The patient was evaluated for edge-to-edge repair of both valves, but because of TR originating from all 3 commissures, the chance of success was low (Figure 1A). Screening for TTVR showed feasible anatomy for an EVOQUE 48 mm (Edwards Lifesciences) via transesophageal echocardiography and computed tomography (Figures 1B to 1D). MR, originating from the medial A2/P2 region, was suitable for M-TEER. The interdisciplinary heart team at the Medical University of Vienna recommended M-TEER and TTVR. The procedure was performed under general anesthesia with continuous invasive hemodynamic monitoring and mild to moderate noradrenalin administration.

**Figure 2:**
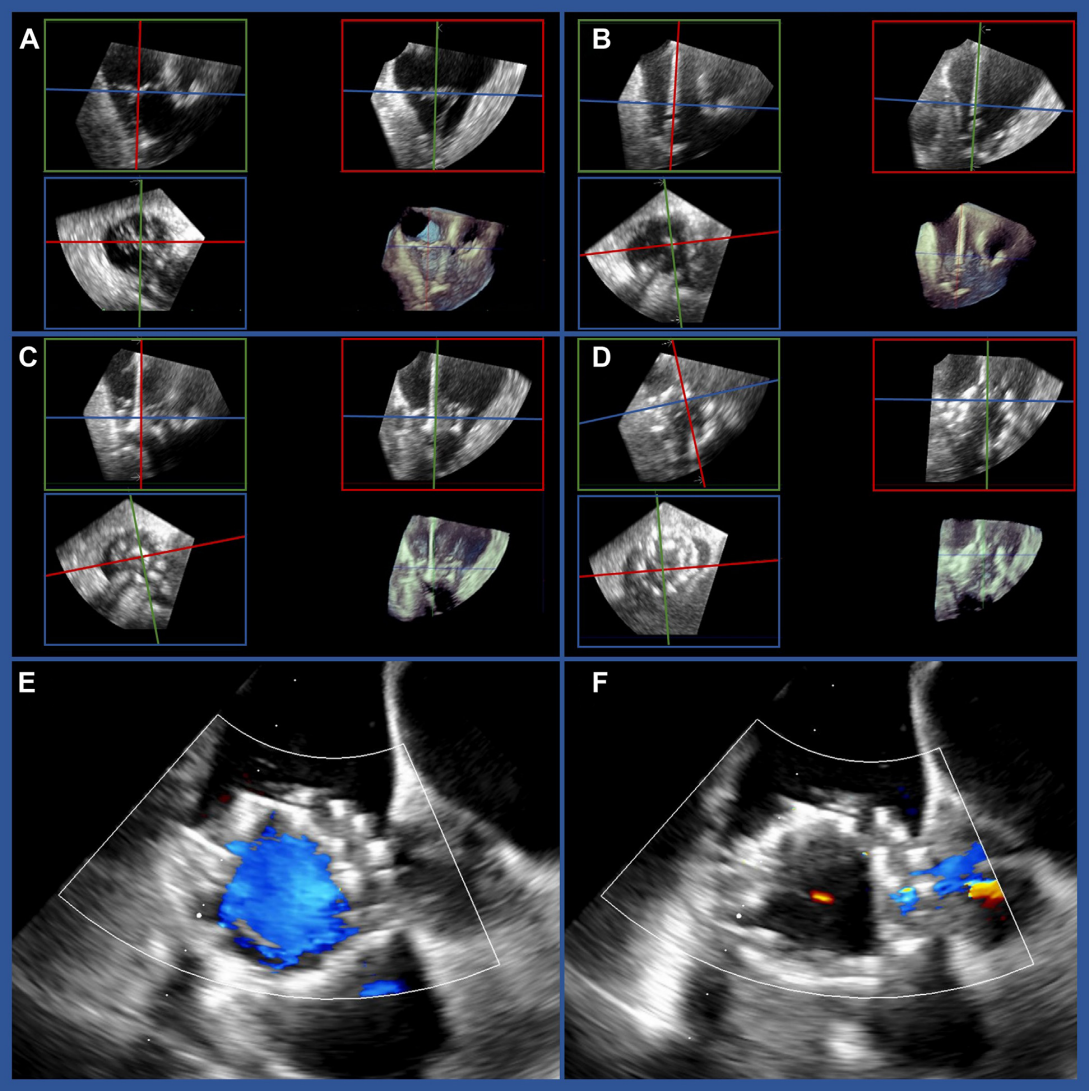
Implantation of the EVOQUE System (A) Initial imaging before implantation of the EVOQUE. (B) Advancing the delivery system. (C) Positioning of the anchors. (D) Full release of the valve and retraction of the delivery system. (E and F) Final result.

The interventional team commenced the procedure with TTVR, preparing large-bore femoral vein access via ultrasound-guided puncture and placement of 2 suture-based vascular closure systems (Perclose ProStyle, Abbott). The 28-F EVOQUE delivery system was successfully introduced into the right atrium and right ventricle. The deep esophageal echocardiographic window showed good imaging quality, and a so-called "home view" was acquired, consisting of the tricuspid inflow outflow view, the grasping view, the short-axis view, and a 3D image using multiplane reconstruction (Figure 2A). To initiate the implantation, a 0.035 preshaped stiff guidewire (Safari XS, Boston Scientific Corporation) was placed in the right ventricular apex through a steerable sheath (Agilis NxT, Abbott). The delivery system was positioned perpendicular to the tricuspid annulus, and the soft atraumatic tip was advanced to expose the delivery guide outlet, creating a so-called “capsule gap” (Figure 2B). The prosthesis was stepwise released until full atrial deployment (Figure 2C). During this process, every anchor was continuously checked for position under the leaflets by “spinning” the echo planes around the short-axis view. In the final step, the valve could be fully released (Figure 2D), and postinterventional inflow showed no stenosis with a mean gradient of 1 mm Hg (Figure 2E) and trace TR (Figure 2F). The system was retracted, and femoral vein access was preserved by placing a 26-F Gore Dryseal sheath.

**Figure 3:**
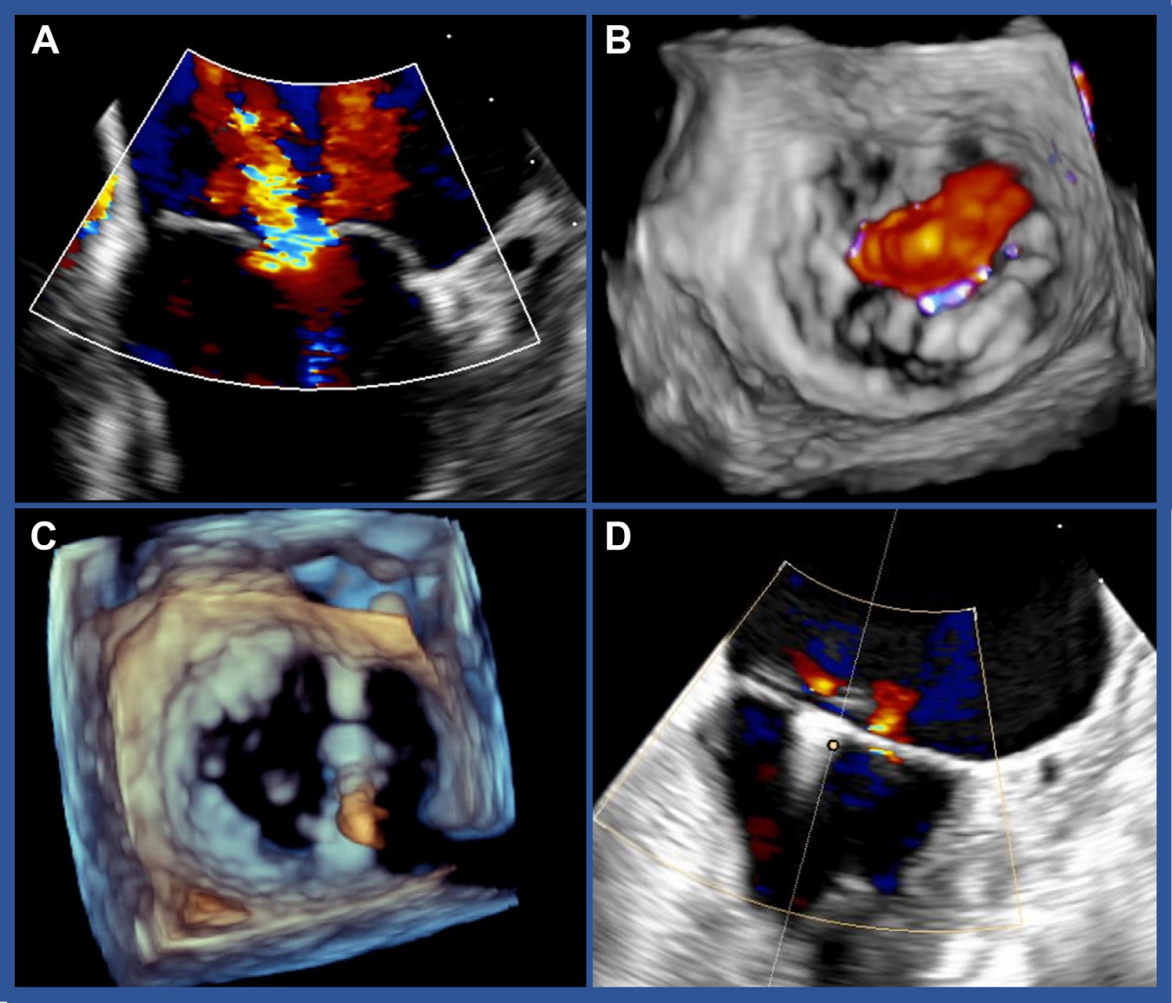
Evaluation and Treatment of Mitral Regurgitation Using a PASCAL Ace Device (A and B) Severe mitral regurgitation according to echocardiography in 2 and 3 dimensions. (C) Implantation of a PASCAL Ace slightly medial. (D) Final result.

MR was re-evaluated, confirming initial screening results (Figures 3A and B). A transseptal puncture was performed, and the system was changed to the 22-F PASCAL guide sheath over a stiff wire placed into a pulmonary vein. Despite introducer downsizing (28- to 26- to 22-F), there was no bleeding, and a PASCAL Ace (Edwards Lifesciences) was successfully placed to connect the medial part of the A2 and P2 segments, reducing MR to mild without significant stenosis (Figure 3D). After retraction of the delivery system, an atrial septal defect with a minor left-to-right shunt remained. The implantation time was 116 minutes. The patient was extubated in the operating room and transferred to the postoperative recovery room, with continued hemodynamic monitoring until the following day, during which Noradrenalin was gradually reduced.

**Visual Summary:**
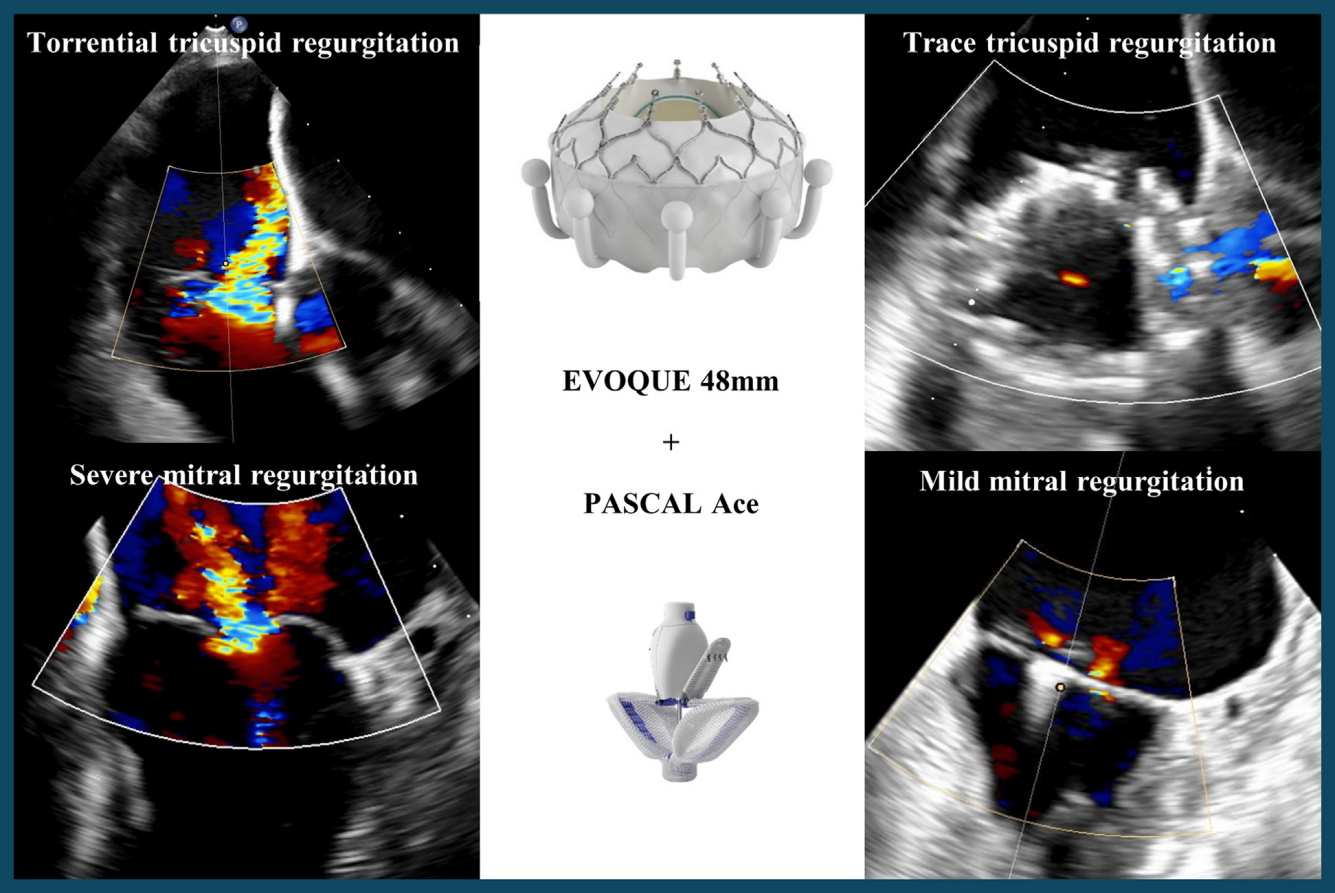
Combined Treatment of Tricuspid and Mitral Regurgitation An 80-year-old woman with severe mitral and torrential tricuspid regurgitation treated with EVOQUE and PASCAL precision.

## Outcome and Follow-up

Postinterventional transthoracic echocardiography revealed well-positioned devices with trace TR, a mean tricuspid inflow gradient of 2 mm Hg, mild MR, and a mitral inflow gradient of 3 mm Hg. Both left and right ventricular function were preserved. Postinterventional electrocardiogram indicated atrial fibrillation with a QRS duration prolongation to 114ms (+18 ms) in a right bundle branch block formation without atrioventricular conduction disturbances. There was no access site bleeding, and anticoagulation with apixaban resumed the day after the procedure. The patient could be discharged 4 days after the procedure.

At 1 month of follow-up, the patient reported symptom improvement, including reduced leg edema and dyspnea (NYHA functional class I-II). Echocardiography showed a persistent reduction in TR and MR to trace and mild, respectively, with no significant stenosis. The QRS duration was prolonged to 124 ms. No serious adverse events were reported.

## Discussion

In this case, we successfully performed a combined TTVR and M-TEER procedure, addressing both regurgitant lesions safely and effectively in under 2 hours.

Transcatheter mitral valve repair is increasingly common, with over 10,000 procedures annually in the United States.^9^ Although T-TEER can effectively reduce TR, it poses challenges in complex anatomies.^6^ The Edwards EVOQUE transcatheter tricuspid replacement system, featuring 9 anchors, overcomes the difficulties of anchoring the new valve in the often enlarged tricuspid valve. The recently published 1-year outcome of the TRISCEND (Transfemoral tricuspid valve replacement and one-year outcomes) trial showed significant improvement in functional status and echocardiographic parameters.^10^

An EVOQUE 48 mm was chosen for our patient to treat TR, and the PASCAL Ace device was favored for the confined mitral valve prolapse. The team also discussed which lesion should be treated first and decided to start with the tricuspid valve. We were concerned that TTVR could lead to some distortion of the basal right ventricular and tricuspid anatomy, which could also affect the mitral anatomy. This could potentially worsen the outcome of an M-TEER procedure. Conversely, M-TEER is very unlikely to affect the outcome of TTVR.

The decision to perform a combined mitral and tricuspid intervention may be questioned based on a recently published study by Adamo et al^5^ in which 35% of M-TEER patients were found to have an improvement in TR of ≥1 degree during short-term follow-up. However, this study did not differentiate between severe, massive, and torrential TR. Our patient had torrential TR, and we estimated the chance of improvement to the non-significant range to be relatively low. Furthermore, the etiology of TR seems to be of atrial origin, most likely because of atrial fibrillation. Both atria were already severely enlarged (Table 1), and reverse remodeling after M-TEER seemed very unlikely. The relatively low pulmonary pressure supports this theory. Therefore, we performed TTVR directly to reduce the risk of worsening TR and right ventricular function, which may lead to higher mortality.

**Table 1:** Patient Details

Baseline characteristics	
Gender	Female
Age, y	80
Dyspnea	NYHA functional class III
Leg edema	Marked
ECG	Atrial fibrillation, QRS 96 ms
Coronary angiography	Normal coronary arteries
Renal function	GFR 68 mL/min
NT-pro-BNP, pg/mL	3,543
Pre-existing conditions	HFpEF, atrial fibrillation, arterial hypertension, COPD, hypothyroidism
Medication	Furosemide, spironolactone, empagliflozin, apixaban, bisoprolol, valsartan, levothyroxine, inhalation therapy
EuroScore-II, %	5.64
Invasive hemodynamics	
Pulmonary artery pressure (systolic/diastolic/mean), mm Hg	42/20/27
Pulmonary capillary wedge pressure (atrial/ventricular/mean), mm Hg	NA/19/20
Right atrial pressure (atrial/ventricular/mean), mm Hg	NA/7/6
Echocardiography	
Tricuspid regurgitation	Torrential
Vena contracta, EROA, RegVol	23, NA, NA
Mitral regurgitation	Severe
Vena contracta, EROA, RegVol	9, 0.4 cm^2^, 63 mL
Right ventricular function	Preserved
TAPSE, TDI s’	19 mm, 10 cm/s
Left ventricular function	Preserved
Ejection fraction, %	56
Left ventricle	Normal size
End-diastolic volume, mL	71
Right ventricle	Normal size
End-diastolic basal diameter, mm	37
Left atrium	Severely enlarged
End-systolic volume, mL	171
Right atrium	Severely enlarged
End-systolic area, cm^2^	31
(Post)interventional details	
Access	Right femoral vein, 27-F
Prosthesis tricuspid	EVOQUE 48 mm
Device mitral	PASCAL Ace
Tricuspid regurgitation	Trace
Mitral regurgitation	Mild
Procedural time, min	116
ECG	Atrial fibrillation, QRS complex 114 ms
Anticoagulation	Apixaban
Mean tricuspid inflow gradient, mm Hg	2
Mean mitral inflow gradient, mm Hg	3

COPD = chronic obstructive pulmonary disease; ECG = electrocardiogram; EROA = effective regurgitation orifice area; GFR = glomerular filtration rate; HFpEF = heart failure with preserved ejection fraction; TAPSE = tricuspid annulus plane systolic excursion; TDI = tissue Doppler imaging.

In the presented patient, we observed a postinterventional right bundle branch block, which was prolonged in QRS duration during the follow-up examination after 1 month. Nevertheless, atrioventricular conduction was adequate. In the recently published TRISCEND study, 13.3% of patients without a pre-existing pacemaker received a new pacemaker after TTVR. Of these 15 patients, 8 had an additional conduction disturbance, including a right bundle branch block.^10^ There is currently no further data on conduction disturbances after TTVR. In particular, risk factors for the need for a permanent pacemaker after the procedure and the influence of dyssynchrony with QRS prolongation on left and right ventricular function should be investigated in more extensive studies in the future.

The total procedure time for both valves was 116 minutes, less than the bypass time of a surgical repair or replacement of the mitral and tricuspid valve, which was a mean of 144 minutes in a recent study.^11^ Furthermore, the 30-day mortality of M-TEER in a large randomized trial was around 2.7%, whereas it was 0% in a randomized TTVR trial.^7^^,^^10^ No data show increased mortality in combined mitral and tricuspid interventions. Still, it can be assumed that the peri-interventional mortality is lower than the calculated EuroSCORE II of 5.64%, which reflects the 30-day mortality of combined mitral and tricuspid surgery.

## Conclusions

We present a rapid and successful treatment of MR and TR with high safety and low interventional risk. We hope we have paved the way for more multivalvular interventional repair and replacement strategies and look forward to future studies that include more patients.

## Funding Support and Author Disclosures

Dr Dannenberg and Prof Bartko have industrial relationships with Abbott and Edwards Lifesciences. Prof Bartunek has industrial relationships with Abbott. All other authors have reported that they have no relationships relevant to the contents of this paper to disclose.

## Appendix

Video 1 Combined Treatment of Tricuspid and Mitral Regurgitation
